# Hospital Reports

**Published:** 1837

**Authors:** 


### WESTERN JOURNAL

OF THE

### MEDICAL AND PHYSICAL SCIENCES.

---

**For October, November, and December, 1837.**

---

### ESSAYS AND CASES.

---

### Art. I.—Hospital Reports.

*Cases treated in the* Cincinnati Hospital; *with Clinical Observations.* Recorded, in part, by Edward Kimball, House Surgeon. Arranged by the Editorial Committee.

#### Fungus Hæmatodes of the Right Elbow.

Samuel Bromley, aged 22, a native of Virginia, and a farmer by occupation, fell upon his right hand in January, 1837, by which he was prevented from working about three weeks. His health had previously been perfectly good; and at the period here specified he resumed his occupation, and remained undisturbed until early in last June, when his attention was directed to the part by a slight pain seated at the bend of the arm, in the course of the ulnar nerve. The pain now became constant and more severe, and was followed, in about twenty days after it was first perceived, by the development of a small lump at the internal condyle of the humerus. As this increased, other nodules appeared at different regions of the elbow, until at length the whole joint was surrounded by them, and became perfectly useless, owing to its great bulk and complete rigidity. He arrived in this city on the 10th of Nov'r, and immediately entered the surgical ward of the

Cincinnati Hospital. The pain in the affected parts, at this period, was very slight; the skin was perfectly natural, excepting that the superficial veins were very much enlarged; the elbow was sixteen inches in circumference, nearly cylindrical in shape, and imparted that peculiar fluctuating sensation to the fingers, which forms so striking a trait of encephaloid disease.

The case having been carefully examined, and its nature ascertained, it became obvious that amputation of the limb would afford the only chance of success. The patient's general health being quite good, the operation was performed on the 11th of November, by Professor McDowell, in the anatomical amphitheatre, in the presence of his colleagues and the medical class. The arm was cut off circularly, nearly midway between the elbow and shoulder, and dressed in the usual manner. Several arteries were, of course, obliged to be tied.

On the 16th, when the stump was first inspected, the lower half of the wound was found to have united by the first intention; the other half being in a state of suppuration. After this, the dressings were applied every third day; the parts continuing gradually to contract adhesions during the patient's sojourn in the hospital. There was little or no febrile excitement, and the only medicines that Bromley took were a small dose of calomel, and an ounce of sulphate of magnesia, to obviate constipation. He left the institution on the first of December, contrary to the advice of the attending surgeon, when the whole of the wound had nearly cicatrized, excepting round the ligatures, none of which had been detached. There was no sign whatever of a reproduction of the disease; but at the parts just mentioned was a discharge of dark-colored sanious matter, probably from the irritation of the protruding threads. How the patient is getting along, we have no information; but we shall endeavor to ascertain this matter, and communicate the results in the next number of the Journal.

The diseased mass having been carefully dissected, and the humeral artery distended with size, and colored with vermilion, was demonstrated by Professor Gross to the medical class. It was of an irregularly lobulated character, of a soft doughy feel, and seemed to spring from the fibrous covering of the humerus, by a broad base, occupying nearly the entire circumference of the bone, as far as its surface of articulation with the radius and ulna. The largest of the lobes were of an ovoidal figure, and nearly six inches in length, by two inches in breadth: the smallest were about the volume of a white walnut. The circumference of the joint, after the removal of the skin and cillulo-adipous tissue, was fourteen inches and a half; the diseased mass being most prominent at the internal condyle. Upon its anterior part reposed the humeral artery and the median nerve, together with the bicipital and anterior brachial muscles, both of them thrust out of their natural position. The muscles connected to the inner condyle were similarly displaced, and several of them were pale, emaciated, and infiltrated with sero-albuminous fluids. The periosteum, where it gave attachment to the morbid growth, was slightly thickened, but otherwise healthy. Externally, the bone was perfectly sound; but internally, for about five inches, its structure was broken down, of a white color, interspersed with reddish spots, and filled with cerebriform matter. A thin capsule of cellulo-fibrous tissue surrounded the diseased mass, and sent processes into its interior, by which cavities were formed, of variable dimensions, for the lodgment of its proper substance. This substance had all the properties of the cerebral texture, being of a light ash color, unctuous to the touch, semi-fluid in its consistence, and of a peculiar spermatic odor.

In exhibiting the tumor to the class, Professor Gross pointed out the difference between scirrhus and encephaloid disease. Hard cancer, he remarked, was seldom witnessed before the age of twenty-five or thirty, whereas the present variety was most common in childhood and adolescence, although cases occasionally occurred in the old and decrepid. The one was soft, semi-fluid, fluctuating, and composed of a substance very similar to that of the brain; the other was more or less dense, gristly, of a pale yellowish, greyish, or brownish color, and intersected by whitish filaments and bands, running in a radiating direction from the centre towards the circumference. Both might occur, he said, in almost every organ of the body; but they originated most frequently in the bones, eye, mamma, testicle, liver, lungs, and subcutaneous cellular tissue. In children, encephaloid disease often occupied the shoulder, the region of the clavicle, the fore-arm or hand; situations in which scirrhus was rarely, if ever, met with. It might exist as a primitive affection; but oftentimes co-existed with hard cancer, tubercles, melanosis, and hydatids.

In their mode of organization, a great difference, the Professor stated, existed between these two heterologous formations. In one, namely scirrhus, the diseased mass was entirely dependent for its vascular supply upon the circumjacent tissues, without whose assistance its growth and nutrition would be instantly suspended; in the other, the cerebriform variety, the blood vessels were much more numerous, of a larger size, more liable to bleed, and were created, so to speak, in the midst of the morbid deposit, from which they gradually extended towards the circumference of the tumor, where they finally anastomosed with the arteries and veins in their immediate neighborhood. Thus, encephaloid had two circulations; one of them proper to the diseased mass; the other common to it and the surrounding structures. Owing to this peculiarity, it grew much more rapidly than scirrhus, sometimes attaining a frightful size in the course of a few months, as in the case of Bromley. Nerves probably existed in greater abundance in this than in the other variety, though it was a remarkable fact that soft cancer was generally much less painful than the hard.

The diagnosis could be determined readily by the consistence, form, situation and history of the tumor. In most instances the surface of the tumor presented a peculiar glossy appearance, and the subcutaneous veins were almost always greatly enlarged and distended; but as these phenomena were witnessed in nearly all cases of chronic swellings, little reliance could be placed upon them when attempting to decide upon the nature of the disease. The pain that attended was extremely uncertain, being sometimes scarcely perceptible, at other times very severe.

The morbid growth, Professor Gross continued, had a tendency, sooner or later, to open and protrude, generally by ulceration, sometimes by sloughing, and occasionally by the formation and bursting of an abscess. The surface thus exposed presented a red, fungous appearance, was extremely sensitive to the touch, very prone to bleed, and constantly bathed with a thin, fœtid, irritating fluid, the quantity of which was usually very profuse. In some cases pure blood was effused, caused by the rupture of some of the vessels, and so copious and obstinate as gradually to destroy the patient. Such sores were always highly disagreeable, and never healed, from the inability of the parts to form healthy granulations. Sometimes the morbid mass sloughed entirely away, as if it had been neatly dissected from its situation; but these cases were rare, and were almost invariably followed by a reproduction of the heterologous substance. Such is a rapid and imperfect outline of the Professor's remarks on the nature of this singular disease. To these we shall add the following extract from Mr. Syme's "Principles of Surgery," the second volume of which has been recently published in England. It has reference, as will be seen, chiefly to the treatment of this frightful malady.

"Encephaloid disease, in its advanced stage," observes Mr. Syme, "is attended with a greenish-yellow complexion, and general emaciation. If allowed to proceed, it sooner or later destroys the patient by gradual exhaustion. The rapidity of its course, like that of hard cancer, is extremely variable, and cannot be foretold according to any data with which we are as yet acquainted. The treatment of this disease is, if possible, still less satisfactory than that of the one before mentioned. All local applications and internal remedies are admitted to be totally useless. The only mode of affording relief is excision; and, owing to the tendency of the morbid action to diffuse itself into the neighboring parts, whatever be their nature or tissue, as well as the taint, or unhealthy disposition of the system, the operations for this purpose are very often followed by relapse. Unless, however, the case does not permit complete ablation of the tumor, or there should be indications of the disease existing in other regions of the body, or the pulse is quickened by the local irritation, in which case, so far as I have observed, recovery never follows an operation, it is the duty of the surgeon to give the patient the benefit of the chance that is thus afforded; and, of course, the sooner that this is done the better, after the nature of the malady is ascertained. The prospect of permanent relief seems most favorable when the disease is seated in the testicle and bones, and most hopeless when the eye-ball is affected."

### Cataract.

Mrs. F., of Indiana, was introduced into the Ophthalmic department, November, 1837. She had grey cataract of both eyes. Her age was 47, her temperament nervo-phlegmatic. She had labored under the disease upwards of two years, and fifteen months before had been operated on with a needle introduced through the sclerotic coat of the left eye. Whether the operator depressed or only broke up the lens could not be known—the appearance of the eye was nearly the same as that of the right, and the amount of light admitted not much greater. Her health was good, and Professor Drake, her pupils having been dilated with extract of belladonna, operated on her in the surgical amphitheatre, in presence of the medical class, the next day after her admission. Presuming the cataract of the left eye to be merely capsular, and that the lens had been displaced or absorbed, he introduced a straight needle through the cornea. The opaque capsule was extremely brittle, and almost entirely disappeared before the touch of the instrument, a small quantity of milky fluid at the same time spreading through the aqueous humor. The pupil imdiately became black, and the needle was withdrawn. In proceeding to operate on the other eye, the Professor remarked, that the lens was undoubtedly there, and although its capsule might be opaque, like that of the eye just operated upon, still the body of the lens might be so likewise. He resolved, therefore, to introduce the needle behind the iris, and thus be able not only to lacerate the capsule, but to break up and depress the lens. But in attempting to do this he was foiled. The needle passed through the lens without depressing it, as it was soft, and he could not direct its point so as to penetrate the capsule. The cause of this, as he stated to the class, was that the instrument had penetrated the sclerotica, so near to the iris, that its point being straight, could not be directed forward into the pupil, and this again arose from the eye being deeply placed in the socket, and from its receding still further, by muscular contraction, at the moment when the point of the instrument made its first approach to the conjunctiva through which it was to pass. Had the curved needle of Scarpa been employed, its point directed forward would have perforated the capsule. No farther efforts were then made.

Great nervous depression, with nausea and vomiting, ensued; and for four or five days the patient was unable to raise her head from the pillow without nausea. Sinapisms were repeatedly applied to the epigastrium, and always with temporary benefit. She also took antispasmodics and narcotics with some advantage; but purging seemed to afford more relief than either, especially to the gastric symptoms. The eyes scarcely reddened, and no perceptible inflammation occurred to either. At the end of a fortnight, it was resolved to operate on the right eye again. The pupil being dilated, the Professor attempted to penetrate the lower and outer part of the cornea with a common sewing needle; but found that it would not pass through. He then took up a Hey's needle, with which he perforated the cornea; but at the moment when it passed through that membrane, the patient, by a spasmodic movement, threw her head back with such sudden force as to cause the assistant to recede, and the needle was drawn out, followed by the greater part of the aqueous humor. The needle was re-inserted through the same orifice, and made to penetrate the capsule, which was opaque and much firmer than that of the left eye. As soon as this was done, the lens, semi-opaque and semi-solid, escaped in quantities round the shaft of the instrument, constituting a kind of extraction by the use of the needle alone. No inflammation whatever followed the operation, and the nervous irritation was considerably less than after the other operation. She had two or three severe attacks in the eye-ball, which were relieved by narcotics. In less than a fortnight she returned home. The sight of the left eye was perfect: that of the right eye appeared to be returning; but the pupil was still partially filled up with the fragments of the capsule, to the removal of which it was hoped the solvent power of the aqueous humor, and the action of the absorbents would be adequate.

The Professor thought this case of value to the students of ophthalmic surgery, as illustrating some of the minor causes of failure in the most simple operations for cataract. The temperament of the patient greatly predisposing her to spasmodic action, and the sunken state of her eye-ball, in fact rendered it injudicious to introduce the needle behind the iris; for after its introduction, the shaft of it was drawn back against the bony margin of the orbit, and the point of it could not be directed forward to destroy the capsule; and the same muscular sensitiveness led to the sudden and unexpected recession of the patient's head in the second operation, withdrawing the eye from the instrument. The Professor observed, that in no department of practical surgery, can small circumstances produce failure more readily, than in operations on the eye; wherefore on proceeding to the performance of the most easy and simple among them, the surgeon should, if possible, reproduce in his mind all the causes of failure that have been known to him. As an example of this prudential care, he cited, with approbation, Mr. Wardrop, the translator of Baron Wenzel's work on the extraction of the cataract, who tells his readers, that he never proceeded to that operation, without first reading over the thirteen rules or steps in the operation which he had himself laid down.

### Sloughing of the Cornea, and Protrusion of the Iris.

In the month of November, Mr. O., a young man from the country, presented himself at the Infirmary, with his left eye in a state of inflammation. He was unable to raise the lid, so that the eye-ball could be inspected, and the muscles were so irritable, that it was almost impossible to accomplish that object. Every attempt of the fingers was productive of a rush of blood to the parts, and a most copious flow of tears. The inflammation had existed for a number of weeks. His general health was good, and the other eye healthy. At length a sight of his eye-ball was obtained, when it was found that a large opening existed in the lower part of the cornea, with an extensive protrusion of the iris. The conjunctiva of the lids was thickened, granulated, and loaded with blood—that of the globe was in a state of congestion.

Prof. Drake observed, that although in ulceration and sloughing of the cornea, constitutional treatment, sometimes mercurial and sometimes tonic, was often required, in this instance the general state of the patient's health was such as to render it unnecessary, and that local applications only could be relied upon. That these should be of two kinds—leeches to the eye-lids externally and internally, and nitrate of silver to the ulcer. As the protruded iris plugged up the orifice and retained the aqueous humor, he made no excision of it; but preferred first to transform ulcerative into adhesive inflammation, and effect a union between the cornea and iris, by the application of the caustic, adding that the applications for these purposes might probably cause sloughing of the projecting portion of the iris. Pencil nitrate of silver was accordingly applied daily, the eye-lids being held open, and water dashed upon the part, after a few seconds, to prevent the spread of the caustic over the conjunctiva. Every other day he was leeched. Under this treatment the inflammation abated kindly; but the iris remained in a state of hernia.

The Professor, finding that it resisted the action of the caustic, at length cut off the protruding part with a pair of curved scissors, and continued the caustic. Very soon afterwards it was discovered, that the cornea had began to cicatrize; and in two or three weeks, a false membrane had formed over the whole aperture, all protuberance being gone. Above, the cornea was transparent; but the pupil was drawn down and closed. At this time, December 25th, the inflammation of the eye is nearly gone, and the patient can raise the upper eye-lid high enough to permit an inspection of the eye. The inordinate secretion of tears has also ceased. This patient was leeched nearly twenty times on the outside and the inside of the eye-lids. On one occasion the leech, instead of fixing on the conjunctiva of the lower lid, siezed on the sclerotica, near its junction with the cornea, and perforating the tunic, drew out great part of the aqueous humor, so that the cornea fell back against the iris. Much ecchymosis of the front of the eye-ball was, also, the consequence of this accident. In two or three days, however, the congestion was gone, and the aqueous humor reproduced.

In reviewing this case, Prof. D. took occasion to express his high opinion of the efficacy of solid nitrate of silver in corneal ulceration, expressing his regret that physicians do not constantly resort to it on the first appearance of ulcerative action in the endemic ophthalmia of this country. He had seen it repeatedly arrest that kind of morbid action, at its commencement, and thus preserve the cornea from perforation, and the iris from protrusion. He also spoke with emphasis of the great value of leeching, in chronic ophthalmia, unattended with constitutional disorder. He would leech, and apply nitrate of silver or the sulphate of copper, at the same time. The former would carry off the local plethora, and the latter, by constringing the vessels, prevent its return.

### Fistula Lachrymalis.

Mrs. T., of the State of Ohio, the subject of this report, was an outstanding patient of the Infirmary. She is about 35 years old, of a nervo-lymphatic temperament, and generally enjoys good health. Almost from her childhood she recollects, that the tears used to collect in the lachrymal sack of the left eye, and that by pressing upon it, they would be made to flow down into the nostril. About the first of October, of the present year, she perceived that she could no longer do this, and at the same time the sack and surrounding parts became inflamed. At the end of a week, during which the swelling and pain constantly increased, she came to Cincinnati to consult Prof. Drake. He found that an abscess was forming, and directed leeches, with the subsequent use of a solution of acetate of lead, which, indeed, she had been already employing. She had no fever. Being under the necessity of returning home immediately, he did not see her again for nearly a month, when she returned with a ruptured abscess on the cheek, below the internal angle of the eye, from which there was a free discharge of tears and pus. Nothing whatever passed down the *ductus ad nasum.* The opening into the abscess was so low down, that neither a probe nor syringe could be made to reach the orifice of the duct, through it, in such a direction as to pass towards the nose. Recourse was then had to the *puncta lachrymalia;* but they were found too small to admit the finest probe. It was necessary then to extend the opening in the abscess up to the sack, which, as the parts were not very sensible, a common circumstance in such diseases, gave very little pain. The foramen could now be reached with facility; but a blunt silver probe could not be passed down the duct, nor any impression made upon it with the syringe. Foiled in these efforts, and unwilling to pierce the *os unguis,* the Professor had recourse to a large, strong surgeon's needle, grasped near its middle, with a pair of forceps. Its curvature favored its introduction, and without much difficulty its blunt end was introduced into the duct. The force that was then found necessary to push it through, was at least equal to what would have been required to push it the same distance into a piece of cork; and the pain which the patient experienced was very great. The passage being opened, a silver pin, with a small head, and slightly curved, which had been used in the operation for hare-lip, was introduced, and its head lodged within the sack. Considerable inflammation followed these violent operations. In two days the pin was withdrawn, covered with pus. At this time Prof. D. had just received, from Dr. McDowell, of Virginia, the interesting practical paper inserted in this number of the Journal, on the use of slippery elm bark in the treatment of diseases of the mucous canals, and among the rest of the *ductus ad nasum,* and he resolved to give the recommendation a trial in this case. Accordingly, a tent or plug, about one line in diameter, swollen into a small head at one extremity, was dipped in warm water for a few seconds, till its surface became soft, and then introduced. When extracted the next day, it had swollen a little, and was covered with pus. The patient found it comfortable, and it was thenceforward the only substance introduced. The external opening, kept covered with adhesive plaster, discharged nothing except when the dressings were removed, nor do the tears flow over the cheek. The abscess granulated rapidly, and it soon became necessary to omit the bougie; to which there seemed no objection; for it was easily introduced, easily extracted, and always came out so covered with *healthy* pus, as to indicate that little danger of a new obstruction existed. After the discontinuance of the bougie, the granulating surface, touched once or twice with sulphate of copper, rapidly cicatrized: but when the patient was about to start home, it was discovered, on pressing the sack, that a drop of tears issued through a very small orifice, which discharged nothing except when such pressure was made. Pointed caustic was immediately applied, and repeated twice; but not producing adhesion, recourse was had to the actual cautery, soon after which the patient was under the necessity of returning home, so that the effects of the application are not now known.

Prof. D. was of opinion, that had he relied on *silver* probes in this case, the obstruction in the duct could not have been overcome. They would all have bent under the force necessary to destroy it, and threw out the opinion that many cases, which have been treated by perforating the *os unguis*, might have been cured by the use of steel probes, slightly curved. Of the value of the elm bougie in this case, he thought there could not be two opinions. He supposed that nothing else could have done so well; and that this valuable native substance, which has not, as yet, ever been accurately analyzed, is applicable to a great variety of useful purposes in physic and surgery, to which it has not been applied.

### Clinical Remarks of Prof. Harrison, on Intermittent Fever and Dysentery.

You will find, gentlemen, that this primary type of fever, intermittent, cannot be made amenable to any exclusive mode of treatment; but that to conduct the treatment judiciously, we must reflect upon, 1st. the general condition of the system; 2ndly, pay attention to the state of the chylopoietic organs, and, 3rdly, weigh well the complications which intermittent fever is liable to induce.

As regards the general condition of the system, we should ascertain whether or not there be an inflammatory or opposite diathesis present. Should there exist any deranged state of the functions of the digestive tube, marked by furred tongue, depraved alvine excretions, or confined bowels, we must rectify such a disordered condition of the abdominal viscera, before we administer quinine. Where there is an enlarged or tumid state of the liver, or an irritated or inflamed condition of the mucous tissue of the intestinal canal, we, of course, should, in our treatment of intermittent have strict reference to such, and other contingent complications. In cases where there exists a positive plethora, or fulness of the vascular apparatus, or where a relative plethora of particular organs is obviously present, as indicated by pain, and a crippled exercise of their appropriate functions, we find it expedient to deplete by general blood-letting, carried to a moderate extent, or by the employment of emetics and purgatives. In a few instances of relative plethora tending to congestive irritation, or painful accumulation of blood, in an organ, such as the brain, or liver, you will find it an expedient preliminary measure, before employing any anti-periodic remedy, to abstract blood locally from the temples by leeches, or cups, or from the region of the liver, by the same process. If inflammation is distinctly indicated in either of the three great splanchnic cavities, we should, by no means, neglect a guarded employment of those depletory measures which will subdue it. After moderate depletion in all such cases of positive or relative plethora, as just referred to, we ought, in the exhibition of quinine for the arrest of the paroxysms of the fever, not lose sight of the modifying agency exerted by combination of medicinal substances of apparently a conflicting mode of action. Thus, there lies a man in that bed, who has been bled, puked and purged; for he had intense pain in the head during the interval of the attacks, accompanied with hot skin and full pulse. After this pretty free depletion, we directed the following prescription:

℞ Quinine grs. iij.
Tart. Emet. grs. ss.
Opium, " ss. M. ft. pil. One to be given every two hours.

This combination, or a similar one, with varying proportions of the constituents, has proved more successful than the tonic plan alone. Sometimes I give a grain of tartarized antimony, or two grains of ipecacuanha, or antimonial powder, with one-third of a grain of morphine with the quinine. This combination has proved more successful in obstinate agues in Italy, than any other means of cure. Calomel may at times be added to the prescription, where there is a failure in the functions of the liver. There is no need, however, of salivation in such cases, unless the hepatic apparatus be considerably deranged. Such derangement being evinced by an enlargement of the organ, by jaundice, or persistent pain in the right hypochondrium.

Where there is not plethora, positive or relative, or inflammation, present in the case, and the primæ viæ are prepared for the reception of our great anti-periodic tonic, especially should the patient be much reduced in strength, as is the precise state of this man, Whitinghall, then we may administer the quinine with a bold hand. This patient took quinine in doses of several grains, for several days; but I found that his ague recurred regularly every day. I then determined to try the effect of large doses; so I directed him to have, in one day, two doses of ten grains each; but still he had a slight chill. I then ordered him two doses, of 20 grains each; one to be given at 11 o'clock A. M., and the other at 1 o'clock P. M., of the same day. These forty grains of quinine, given alone, broke, as we familiarly say, his ague; and he has remained well. Now, I wish you to ask this man about the sensations which he experiences in his ears. Upon inquiry, he will tell you, that he has a severe, heavy pain, penetrating both ears, seeming to pass through the petrous portions of the temporal bones. And this pain of the ears, with diminished capacity of hearing, has resulted from the large doses of quinine which he has taken. I have never found any phenomena present in such cases which would lead my mind to the inference, that this disturbance of sensation and function in the auditory apparatus was the product of any tendency to inflammation. I believe this singular effect of the remedy entirely a neuralgic phenomenon; and this is all I know about it. It passes off in a few days, without any impairment of hearing.

I wish, young gentlemen, to guard your minds from a too common error, entertained on the subject of one of the complications of ague and fever. I refer to an enlarged state of the spleen, commonly called by the people "ague cake." Twining, a late author, who practised, with distinguished ability, for some years, in the East Indies, has cautioned us against the excessive use of mercury, in diseased spleen, accompanying or succeeding intermittent fever. And I would reiterate the language of monition uttered by him, and say to you, beware how you attempt to cure enlarged spleen by salivation, especially in such cases as are affected with a "splenic cachexia," as the author denominates that enfeebled state of the constitution, which is often the accompaniment of splenic enlargement. The most successful mode of conducting the treatment of such cases is, after arresting the ague, to employ a combination of aloes and iron, as in this formula:—take of aloes six grains, sulphate of iron four grs., and garlic twenty grs., made into four pills, which are to be given during the day. Or we may employ this formula:

℞ Jalap, Rhubarb. Columba,

Ginger, and Crem. Tart., aa ℥i.

Ferri Sulphas grs. x.

Tinct. Sennæ ℥iv.

Peppermint Water ℥x. M.

The dose of this mixture, recommended by Dr. Twining, in his Clinical Illustrations of the Diseases of Bengal, for enlarged spleen, is one ounce, twice a day. Should it purge too actively, half the quantity may be directed.

But, gentlemen, let us turn our attention to another form of disease, which is frequently met with in the practice of medicine. Here lies a man with severe symptoms of dysentery. He has tormina, tenesmus, and bloody evacuations from the bowels; besides, this poor fellow has experienced a loss of blood by vomiting, amounting, Mr. Kimball says, to a pint. The hæmatemesis occurred in the night, and the blood thrown up was of a dark grumous character. No doubt, it took place by exhalation, and not by rupture. This sanguineous emission from the upper portion of the alimentary tube, will, I think, act remedially. This patient has, according to my views of the pathology and cure of dysentery, taken too much morphine, before his admission into our hospital. What is the pathology of dysentery? Certainly, it is essentially a disease of inflammation. Of what part of the intestines? Of the colon and rectum; the mucous coat of both these bowels being inflamed in acute forms of the affection, and in chronic dysentery ulceration most frequently supersedes.

But dysentery is modified in its character by its etiology, the constitutional powers of the patient, and various other modifying circumstances. Still it retains some of its original features. It is an inflammatory disease, and requires a treatment in accordance with such a pathological state. Understand me not as recommending an indiscriminate depleting mode of treatment for all cases of dysentery. I do not believe in any abstractions in the practice of medicine. All cases of disease are ever varying modes of abnormal vital manifestation. All *a priori* conceptions of disease are to be banished from the bed-side, nor are we to be misled by the name of inflammation, when applied to a disease. Inflammation is no more a unit, one and the same and indivisible, than is fever. When, therefore, we urge that the true pathological character of dysentery is inflammation of the lower bowels, we are not to be understood as asserting that the disease can be cured precisely as we cure an inflammation of the lungs, or of the brain. Our treatment must undergo necessary modifications, in order to fulfil those contingent indications, which are so apt to arise in the progress of the disease. Besides, it is of primary importance for us to keep in view this important truth—that an inflammation in a mucous tissue cannot be treated precisely like an inflammation in a serous tissue, or in the parenchymatous structure of the organs. Dysentery from miasmata demands a treatment somewhat different from a dysentery resulting from mere vicissitude of atmospheric temperature. In bilious or hepatic dysentery, of a miasmatic origin, after sanguineous evacuation, we employ calomel in scruple doses, aided by castor oil, until we elicit a free discharge of bile. In a case of dysentery arising from cold applied to the surface of the body, vascular depletion may be carried to a greater extent, both generally and locally, than in dysentery from hepatic derangement. Opium, or the salts of morphia, are frequently employed too early in dysentery. But we cannot dispense with the invaluable soothing action of this great anodyne in the latter stages of the disease. Ipecacuanha, with opium, has been exhibited with decided curative results in dysentery, in both its acute and chronic forms. From five to twenty grains of ipecacuanha are administered once or twice every twenty-four hours, in conjunction with one or two grains of opium. The Dover's powder, as you know, contains equal parts of ipecac and opium, and therefore cannot be so advantageously employed in the first stage of the disease as a combination of the same constituents of the prescription, given in different proportions.

Where a patient is affected with dysentery and ague at the same time, we should, after due depletion by the lancet, or by a local depletion of blood from the abdomen, proceed to arrest the paroxysms of the intermittent. To this end we give the following:

℞ Calomel ℈j.
Ipecacuanha grs. x.
Sulph. Quinine grs. xij.
Opium grs. iv. M. Divide into four pills.

One of these pills is to be given every two hours, so as to have the whole of them administered at least half an hour before the anticipated seizure of the ague, or chill. Allowing a few hours to elapse, or after the usual period of the paroxysm has passed, we give a dose of castor oil, to facilitate the operation of the calomel on the secretions of the liver and mucous tissue of the great intestines. The diet in dysentery should consist of the mildest and most unirritating substances, taken in a fluid form, and the patient should never be allowed any solid aliment, in such cases, even during convalescence, as the fœcal contents of the colon, which are the result of solid alimentary materials, will renew, or greatly exacerbate the irritation of the bowel. Rice, gum arabic, slippery elm, Irish moss, &c., are the most eligible substances to be used by the patient in a state of weak solution, and drank tepid, in moderate quantities, at a time.

Here you have a case of intermittent, complicated with acute pain in the left side; which I consider to be an affection of the spleen; perhaps some degree of splenitis. Of this, however, I am not certain; for inflammation of the spleen is of very infrequent occurrence. Whilst I attended on the Marine Hospital, in Louisville, Ky., a case occurred of rupture of a vessel in the spleen, which caused the death of the patient, by a large effusion of blood into the cavity of the abdomen. The man had been intemperate, and was subject to ague and fever. In one of the paroxysms of ague he suddenly died. The immediate cause of his sudden death was discovered, upon a post mortem examination, to be this splenic hemorrhage. The spleen seems to act in a manner auxiliary to the liver, in preventing those dangerous consequences which threaten that great transmitting and secreting organ in every great inequilibrium of the circulation.

